# Supplementary material for: Whole-Genome Sequencing of Staphylococcus aureus and Staphylococcus haemolyticus Clinical Isolates from Egypt
Source: Microbiol Spectr. 2022 Jun 21;10(4):e02413-21. doi: 10.1128/spectrum.02413-21 (PMC9431571; doi:10.1128/spectrum.02413-21)
Supplement: Supplemental file 1 — Supplemental material. Download spectrum.02413-21-s0001.pdf, PDF file, 0.4 MB [file spectrum.02413-21-s0001.pdf]

Phylogenomic study of Staphylococcus aureus and

Staphylococcus haemolyticus clinical isolates from Egypt

Cesar Montelongo, Carine R. Mores, Catherine Putonti, Alan J. Wolfe, Alaa Abouelfetouh

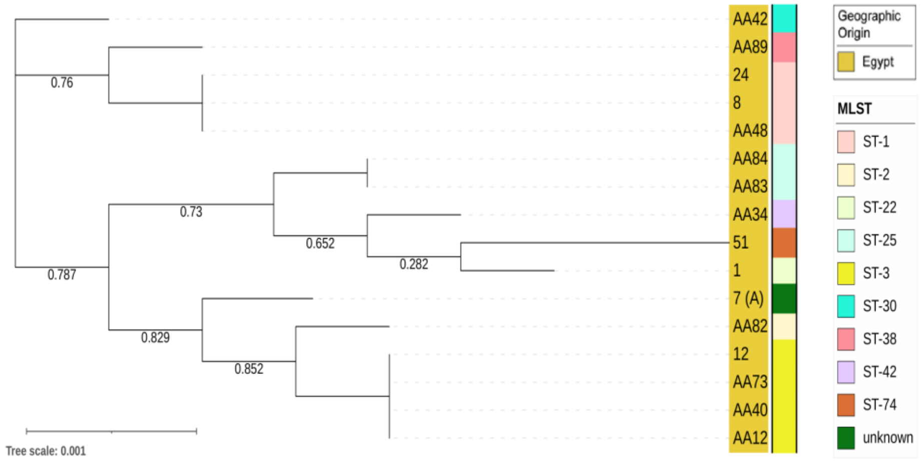

Figure S1. MLST tree of *S. haemolyticus* annotated by geographic origin and MLST.

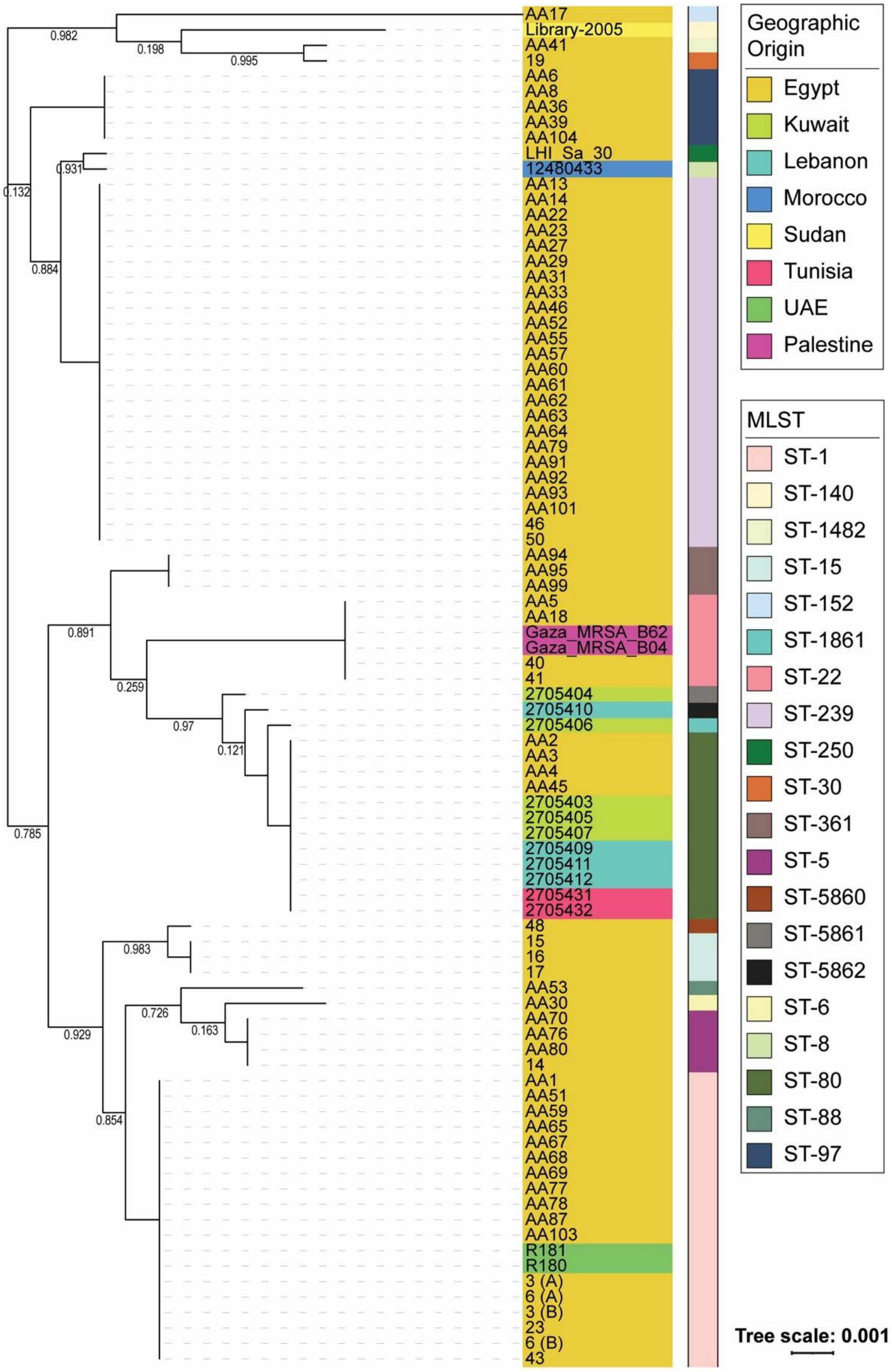

Figure S2. MLST tree of *S. aureus* annotated by geographic origin and MLST.
